# Persistence and Recovery of Polystyrene and Polymethyl Methacrylate Microplastic Toxicity on Diatoms

**DOI:** 10.3390/toxics13050376

**Published:** 2025-05-06

**Authors:** Chongchong Meng, Huijie Yang, Yuan Du, Xiaokang Li

**Affiliations:** 1 School of Environmental and Material Engineering, Yantai University, Yantai 264005, China; 2 Key Laboratory of Molecular Pharmacology and Drug Evaluation, Ministry of Education, Collaborative Innovation Center of Advanced Drug Delivery System and Biotech Drugs in Universities of Shandong, School of Pharmacy, Yantai University, Yantai 264005, China

**Keywords:** microplastics, diatom, nitrogen assimilation, environmental risks, recovery

## Abstract

The increasing pollution of polystyrene (PS) and polymethyl methacrylate (PMMA) microplastics (MPs) has become a global marine environmental problem. Diatoms contribute nearly 40% of marine primary productivity and shape the nitrogen cycle in the oceans. However, the persistence of the phytotoxicity of MPs on diatoms, especially nitrogen assimilation, remains largely unknown. To examine the persistence of PS and PMMA toxicity in diatoms, two subexperiments (a 96 h exposure followed by a recovery phase) were conducted on *Thalassiosira pseudonana* at concentrations ranging from 0.001 to 1 mg/L. The results showed that PS and PMMA inhibited algal growth by 3.76–6.49% and 4.44–8.37%; increased oxidative stress by 10.06–30.51% and 30.46–38.12%; and caused ultrastructural damage by 14.24–25.56% and 12.28–20%, respectively, consistent with the downregulation of glyoxylate, dicarboxylate metabolism, and glutathione metabolism. At the recovery stage, the algal density induced by PS was significantly recoverable at 0.001 and 0.01 mg/L, consistent with the enhanced carbohydrate metabolisms. After recovery, the cell permeability and reactive oxygen species (ROS) levels induced by PS and PMMA were significantly decreased at 1 mg/L, respectively, which was closely related to the downregulation of glycine, serine, and threonine metabolism and the upregulation of pantothenate and coenzyme A biosynthesis. Moreover, the inhibition of nitrogen assimilation enzymic activities induced by PS and PMMA was significantly recovered at 1 mg/L despite the downregulation of nitrogen metabolism. This study highlights the phenomena and mechanisms of phytotoxicity and recovery, and provides new insights for comprehensive understanding and evaluation of environmental risks of MPs.

## 1. Introduction

Since the 1950s, plastics have been widely used in production and people’s lives due to their advantages of convenient processing, high stability, and low price [1,2]. The global yield of plastics was 400.3 million metric tons by 2022, and is predicted to increase by approximately 47% by 2050 [3]. Plastic products break down into fragments through physical, chemical, and biological processes, resulting in a dramatic increase in the amount of plastic waste entering the aquatic and terrestrial environments [4,5]. Currently, large amounts of microplastics (MPs) have been detected in aquatic ecosystems such as the ocean, rivers, and lakes; they have even been detected in polar regions with little human activity [6,7,8,9]. MPs have existed persistently for centuries due to their exceptional stability, posing a significant marine environmental accumulation challenge [10].

Traditional MPs are extremely difficult to degrade in the environment and accumulate in organisms, inducing biotoxicity or environmental pollution [10,11]. Phytoplankton, as primary producers, play key roles in marine ecosystems [12,13]. Studies have shown that traditional polystyrene (PS), polyethylene (PE), and polyvinyl chloride (PVC) MPs could pose growth inhibition, photosynthesis reduction, and oxidative stress to freshwater and marine algae [14,15,16,17,18,19]. Nitrogen assimilation and fixation provide bioavailable nitrogen (e.g., ammonium salt) for the ecosystem [20]. Nitrogen assimilation mechanisms in diatoms play a vital role in the marine nitrogen cycle, which can be affected by different types of MPs [21]. It has been found that PS MPs inhibited nitrogen assimilation under low nitrogen levels while enhancing nitrogen metabolism under high nitrogen levels in *Phaeodactylum tricornutum* [22]. The regulation of gene expression and metabolic processes by PS MPs underscored this result. However, the persistence and recovery of traditional MPs on the nitrogen assimilation of diatoms are still poorly understood.

Moreover, to solve the problem of traditional plastics being difficult to degrade and causing a persistent environmental impact, an increasing number of biodegradable plastics have been developed. However, biodegradable MPs still pose a potential risk to the environment [23]. According to previous studies, biodegradable polylactic acid and polycaprolactone MPs inhibited the growth of marine and freshwater algae and caused oxidative stress [19,24]. Nevertheless, the toxicity of biodegradable MPs, and, in particular, whether nitrogen assimilation is reversible, remains largely unknown. Therefore, exploring the persistence and recovery of environmental toxicity from traditional and biodegradable MPs is necessary to comprehensively assess their ecological risks.

Diatoms are important biological carbon pumps in the oceans, carrying out photosynthesis and nitrogen assimilation processes and contributing nearly 40% of marine primary productivity [25,26]. *Thalassiosira pseudonana* (*T. pseudonana*), a typical diatom, is often used as a model organism for studying MP toxicity [27,28,29,30]. It is reported that MP pollution in seawater measures up to mg/L [31]. PS and polymethyl methacrylate (PMMA) are the common polymer types, and micro-sized PS and PMMA contaminants have been widely detected in marine environments [32,33,34]. Herein, the persistence and recovery of algal growth inhibition, oxidative stress responses, and nitrogen assimilation process induced by PS and PMMA at environmentally relevant concentrations (0.001–1 mg/L) on *T. pseudonana* are studied. The specific toxicological mechanisms of MPs after exposure and recovery are explored in conjunction with metabolomics. The results of this study demonstrate the persistence of toxicity and biological recoverability associated with traditional and biodegradable MPs, addressing the knowledge gap in understanding the effects of MPs on the biological nitrogen cycle, and providing new insights into the overall toxicity of MPs.

## 2. Materials and Methods

### 2.1. Characterization of MPs

PS and PMMA MPs (200 nm) were obtained from Haian Zhichuan Battery Materials Tech Co., Ltd., Changzhou, China. The morphology and size of MPs were examined by sticking the samples on conductive tapes and observing them using scanning electron microscopy (SEM, TESCAN MIRA LMS, Brno-Kohoutovice, Czech Republic). The dried MPs were mixed with spectrally pure potassium bromide to make a pressed sheet, and the specific chemical groups of samples were measured using a Fourier transform infrared (FTIR) spectra instrument (Thermo Scientific Nicolet iS20, Waltham, MA, USA) from 4000 to 400 cm^−1^ at a resolution of 4 cm^−1^ [35]. The zeta potential of MPs at a nominal concentration of 1 mg/L dispersed in pure water with the value of pH 6, 8, and 10 was measured using a Malvern Zetasizer Nano ZS90 (Malvern Instruments, Worcestershire, UK) to examine their dispersion stability.

### 2.2. Exposure and Recovery Experiments

*T. pseudonana* (GY-H27) was obtained from Shanghai Guangyu Biotechnology Co., Ltd., Algae Culture Collection, Shanghai, China. The experiment was conducted on an aseptic operation table. All culture consumables were sterilized and irradiated with ultraviolet light to ensure a sterile environment. Since ISO 10253 does not include *T. pseudonana*, the culture conditions and growth inhibition test were partially adjusted based on the ISO 10253 technical guide to be more suitable for *T. pseudonana* [36]. The details are as follows: the diatom was cultivated in artificial seawater with F/2 medium in 250 mL flasks, with a pH of approximately 8.0 and a salinity of 30‰ [37]. The components of the F/2 medium are given in Table S1. The temperature in the light incubator was set to 20 °C with an illumination of 3000 lux and a 14:10 h light–dark cycle [35]. The flasks were shaken 3 times a day, and the position of the flasks randomly changed every other day to ensure the same culture status of diatoms. The diatoms were cultured for 4 days to adapt to the environment before formal experiments were conducted. The count of algal cells was performed using a plankton count box under a microscope magnification of 160 times, and then the algal density was measured using a spectrophotometer at 685 nm using the following formula: y = 308.8x − 0.27 (Figure S1). Here, y denotes the algal density (10^4^ cells/mL) and x denotes the algal supernatant absorbance value.

Furthermore, the environmental concentrations of MPs in marine waters have been reported to range from µg/L to mg/L, such as 0.148 µg/L in the Atlantic Ocean surface water and 0.62 mg/L in the Colombian Caribbean coastal waters [31,38,39]. Therefore, the algal experiments at MP nominal concentrations of 0.001, 0.01, 0.1, and 1 mg/L were conducted. Lower concentrations (0.001 and 0.01 mg/L) represent common concentrations in marine environments, while higher concentrations (0.1 and 1 mg/L) represent an increasing presence of MPs in the future. And, considering the complexity of MPs in the environment, MP exposure was simplified using PS and PMMA of 200 nm spheres to better compare our findings with other studies. The algal culture without MPs was served as the control, and no additional reference toxicant control tests were performed. The initial density of algae was 2.56×10^5^ cells/mL. To ensure comparability with previous experimental results, and to prevent excessive algal density from affecting algal growth, the experiments were conducted for 96 h under conditions commonly used in algal toxicity research [40,41]. After 96 h of MP exposure, algal cells were washed with fresh F/2 medium by centrifugation at 3500 g for 10 min and collected for further experiment. The nonlinear curve fitting (logistic model) of the growth inhibition rate of microalgae at 96 h was conducted using Origin Version 2025 software, and the effect concentration at 50% growth inhibition (EC_50_) was obtained. For the recovery stage, the collected algal cells were diluted to 1.45 × 10^5^ cells/mL and cultured in fresh F/2 medium. After cultivation for 96 h, algal cells were collected, centrifuged, and washed twice with phosphate-buffered saline (PBS) for the next toxicity index determination. The cell suspension (10 mL) was centrifuged (1433× *g*, 15 min) to obtain the supernatant, and the filtrate was obtained using a syringe with a 0.22 µm aqueous filter for further extracellular ion determination.

### 2.3. Measurements of the Photosynthetic Pigment Contents

The contents of chlorophyll a (Chl a), chlorophyll c (Chl c), and carotenoid were determined using a UV-VIS spectrophotometer (TU-1901, Purkinje General Instrument, Beijing, China). Algal suspensions (2 mL) after exposure and recovery were collected and centrifuged at 6000× *g*, at 4 °C, for 10 min. Then, methanol (2 mL) was added to the cell precipitation, and the mixture was incubated in the dark at 4 °C for 24 h. The supernatants were collected after centrifugation and were subjected to absorbance measurements at 480, 510, 632, 665, and 750 nm. The contents of Chl a, Chl c, and carotenoid were calculated according to the following formula [42,43]:

Chl a (μg/mL) = 13.2654 × (A_665_ − A_750_) − 2.6839 × (A_632_ − A_750_) (1)

Chl c (μg/mL) = −6.0138 × (A_665_ − A_750_) + 28.8191 × (A_632_ − A_750_) (2)

Carotenoid (μg/mL) = 7.6 × ((A_480_ − A_750_) − 1.49 × (A_510_ − A_750_)) (3)

A_x_ represents the absorbance value of the supernatant at the wavelength of x.

### 2.4. Oxidative Stress Responses to MP Exposure

The level of intracellular reactive oxygen species (ROS) was detected using the fluorescent probe 2′,7′-dichlorofluorescein diacetate (DCFH-DA) [35]. Nonfluorescent DCFH-DA that entered the cells can translate into the strongly fluorescent product dichlorofluorescein (DCF) through the processes of hydrolysis and oxidation. The intracellular fluorescence intensity of DCF can reflect the total ROS content in cells. The algal cells were incubated with DCFH-DA (10 μM) at 20 °C in the dark for 30 min and washed twice with F/2 medium. The fluorescence intensity of supernatants was determined using a microplate reader (SpectraMax iD3, Molecular Devices, San Jose, CA, USA) with an excitation wavelength of 485 nm and an emission wavelength of 530 nm. The relative ROS levels of the treatment groups were expressed as the fluorescence intensity divided by that of the control groups and then multiplied by 100%.

Cell permeability was measured using the fluorescein diacetate (FDA) [35]. Nonfluorescent FDA enters the algal cells and is hydrolyzed into fluorescein, which has detectable fluorescence that can be utilized for analysis of cell permeability. The algal cells were incubated with FDA (10 μM) at 20 °C in the dark for 30 min and washed twice with F/2 medium. The fluorescence intensity of supernatants was determined using a microplate reader (SpectraMax iD3, Molecular Devices, San Jose, CA, USA) with an excitation wavelength of 485 nm and an emission wavelength of 521 nm. The relative cell permeability of the treatment groups was expressed as the fluorescence intensity divided by that of the control groups and then multiplied by 100%.

A mitochondrial membrane potential assay kit (PH1787, Scientific Phygene, Fuzhou, China) was used to analyze the mitochondrial membrane potential of algal cells. The algal cells were incubated with tetrachloro-tetraethyl benzimidazol carbocyanine iodide (JC-1, 10 μM) at 20 °C in the dark for 30 min and washed twice with PBS. The fluorescence intensity of supernatants was determined using a microplate reader (SpectraMax iD3, Molecular Devices, San Jose, CA, USA). The excitation and emission wavelengths of green light were set at 490 and 530 nm, respectively, while the excitation and emission wavelengths of red light were set at 525 and 590 nm, respectively. The results of the treatment groups were expressed as the ratio of red-green fluorescence intensity (I590/I530) divided by that of the control groups and then multiplied by 100%.

The activity of superoxide dismutase (SOD) was measured with an SOD test kit by the water-soluble tetrazolium salt (WST-1) method (BC5165, Solarbio, Beijing, China). Superoxide anion (O_2_^−^) reacts with WST-1 to form a water-soluble yellow substance, while SOD can clear O_2_^−^, and thus inhibited the reaction [44]. The shade of color of the reaction solution can reflect SOD activity. The activity of catalase (CAT) was measured with a CAT test kit using the ammonium molybdate method (BC4785, Solarbio, Beijing, China). Hydrogen peroxide (H_2_O_2_) reacts with ammonium molybdate to form a stable yellow complex. By measuring the amount of H_2_O_2_ that remained in the reaction system, the amount of H_2_O_2_ catalyzed by CAT was obtained, and the activity of CAT was reflected [45].

### 2.5. Cellular Ultrastructure Damage

The algal cells were fixed with 2.5% (*w*/*v*) glutaraldehyde at 4 °C overnight, washed with PBS 3 times, and fixed with 1% osmium tetroxide for 2 h. Then, the sample was dehydrated with 30%, 50%, 70%, 80%, 90%, 95%, and 100% ethanol for 15 min, respectively, and finally fixed with dehydrated ethanol and dried using a critical point dryer (CPD 300, Leica, Germany) [46]. A thin layer of gold was deposited on the surface of the sample, and then the cells were observed using SEM (JSM-IT800, JEOL, Tokyo, Japan).

The algal cells were fixed with 1% osmium tetroxide for 2 h and washed 3 times with PBS. Then, the sample was dehydrated with ethanol (30%, 50%, 70%, and 80%) and treated with acetone (90% and 95%) for 15 min, respectively, followed by dehydration in pure acetone for 20 min. The sample was embedded in the embedding agent Spurr and acetone (*v*/*v*; 1:1 for 1 h, 3:1 for 3 h, and 1:0 for 24 h), then polymerized for 24 h at 70 °C. Ultrathin Sections (70–90 nm) of algae were obtained using an ultramicrotome (UC7, Leica, Wetzlar, Germany) and stained with uranyl acetate and lead citrate for 10 min [35]. Cellular ultrastructure damage was observed by transmission electron microscopy (TEM, H-7650, Hitachi, Tokyo, Japan).

### 2.6. Nitrogen Assimilation Measurements

The protein content was determined with a protein quantitative assay kit (A045-4, Nanjing Jiancheng, Nanjing, China). The activities of nitrate reductase (NR), glutamine synthetase (GS), and glutamate synthetase (GOGAT) were determined using the NR assay kit (BC0085), GS assay kit (BC0910), and GOGAT assay kit (BC0075), respectively, provided by Beijing Solarbio Science & Technology Co., Ltd., Beijing, China. The nitrite reductase (NiR) activity was determined with an NiR kit (G0408F48) provided by Suzhou Grace Biotechnology Co., Ltd., Suzhou, China.

The alterations of nitrate content in the medium and intracellular nitrate content were detected with a nitrate assay kit (G0426W, Suzhou Grace, Suzhou, China) by the UV absorption method according to the manufacturer’s instructions. The content of nitrite in the medium and cells was determined by using the nitrite content assay kit (BC1485, Solarbio, China). According to the manufacturer’s instructions, under acidic conditions, nitrite reacts with p-aminobenzene sulfonic acid to form a diazo compound, and then with N-1-naphthyl ethylenediamine to form a magenta azo compound with a characteristic absorption peak at 540 nm. The intracellular ammonium nitrogen content was measured using the ammonium nitrogen test kit (G0410F, Suzhou Grace, Suzhou, China). Following the manufacturer’s instructions, the reduced ninhydrin, ammonia, and ninhydrin hydrate react to form a bluish-purple substance in the weak acid environment with a characteristic absorption peak at 570 nm.

### 2.7. Metabolomics Analysis

The algal suspension (20 mL) was centrifuged (5733× *g*, 15 min) and washed with PBS. The cells were quickly frozen in liquid nitrogen, and the metabolites were extracted with 4 mL of precooled methanol–chloroform–water (volume ratio = 2.5:1:1) solution, with ribitol (100 mg/L, 50 μL) used as an internal standard. The mixture was completely broken with an ultrasonic cell disruptor (Scientz-48, Ningbo Scientz, Ningbo, China) at 300 W for 4 min in an ice-water bath, followed by an ultrasonic cleaning instrument (KQ-200KDE, Kunshan Ultrasonic Instrument, Suzhou, China) at 200 W for 30 min. Subsequently, the mixture was centrifuged (5733× *g*, 15 min) to collect the supernatant. The metabolites in the precipitate were extracted again with the precooled extract solution. The supernatant collected twice was mixed, added with 1 mL of ultrapure water, and centrifuged at 5733× *g* for 15 min. The chloroform and methanol/water phases were stratified. The lower phase (chloroform) was filtered through a 20 mL silica gel column and dried by nitrogen. The upper phase (methanol/water) was filtered through a 0.22 μm polytetrafluoroethylene (PTFE) filter and mixed with the treated lower phase. The mixture was blown with nitrogen and then freeze-dried. The treated samples were derivated with O-methoxamine hydrochloride (20 mg/mL, 50 μL) and N-methyl-N-(trimethylsilyl) trifluoroacetamide (MSTFA, 80 μL) at 37 °C for 90 and 30 min, respectively. The metabolites were detected using gas chromatography–mass spectrometry (GC-MS, 6890A/5977A, Agilent, Santa Clara, CA, USA) in full scan mode with a detection range of *m*/*z* 70–600 [47]. The metabolites were identified according to the National Institute of Standards and Technology (NIST 14.0) mass spectrum library in ChemStation G1701DA software, and the data were analyzed using MetaboAnalyst 6.0 (http://www.metaboanalyst.ca, accessed on 20 August 2024). Differentially expressed metabolites (DEMs) were selected based on a fold change ≥ 1.5 or ≤0.67.

### 2.8. Statistical Analysis

All experiments were conducted at least in triplicate, and the results are presented as the means ± standard deviations. One-way analysis of variance (ANOVA) and the Duncan test were applied to analyze the statistical significance. All statistical analyses were conducted using IBM SPSS 26, and a *p*-value less than 0.05 was considered statistically significant.

## 3. Results and Discussions

### 3.1. Persistence and Recovery of Growth and Photosynthesis to MPs

**Figure 1:**
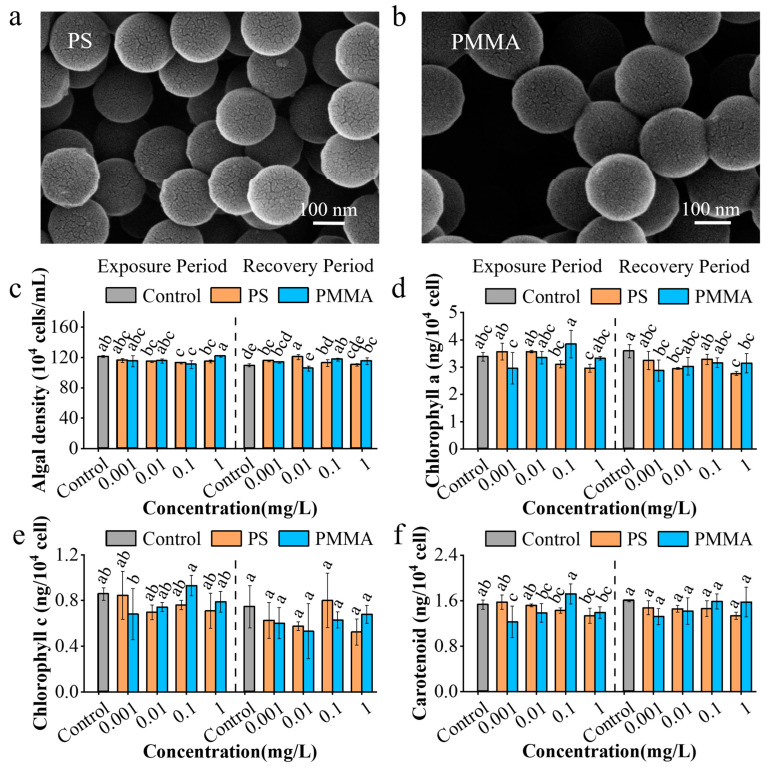
Characterization of MPs and algal growth. SEM images of (**a**) PS and (**b**) PMMA. (**c**) Density of algae. (**d**) Chl a content. (**e**) Chl c content. (**f**) Carotenoid content. Both exposure and recovery experiments were conducted for 96 h. The different lowercase letters in the figure denotes significant differences, *p* < 0.05.

As shown in Figure 1a,b, both PS and PMMA exhibited spherical shapes, with a particle size of 203.7–220.8 nm for PS and 192.3–214.2 nm for PMMA. The FTIR spectra of PS (Figure S2a) showed that there was a C-H-typical absorbance peak at 3025.9 cm^−1^, and a C-C-typical absorbance peak at 1493.0 cm^−1^, indicating the aromatic hydrocarbons on its surface [48]. The FTIR spectra of PMMA (Figure S2a) showed that there was a C=O typical absorbance peak at 1730.7 cm^−1^. A C-O typical absorbance peak was shown at 1269.0 cm^−1^, indicating the ester groups on its surface [48]. The zeta potentials of PS were −13.0, −11.2, and −43.3 mV, respectively, in the water of pH 6, 8, and 10 (Figure S2b). The zeta potentials of PMMA were −10.6, −9.0, and −47.5 mV, respectively, in the water of pH 6, 8, and 10 (Figure S2b), suggesting the similar dispersivity of two MPs.

Compared with the control, PS and PMMA inhibited algal growth by 2.43–13.03% and 1.63–11.65%, respectively, before exposure for 96 h (Figure S3a–c and Figure 1c). The EC_50_ of PS and PMMA after 96 h exposure was 0.01 and 0.1255 mg/L, respectively (Figure S3d,e), indicating stronger growth inhibition of PS than PMMA. As shown in Figure S3f, PS and PMMA did not show significant growth inhibition at 24 h of recovery due to consistent initial algal densities during the recovery phase. However, the onset of algal density inhibition (1.6–8.01% and 2.43–6.85%, respectively) occurred after recovery for 48 and 72 h by PMMA, and PS still did not cause significant growth inhibition in Figure S3g–h, suggesting the easier recovery of growth inhibition induced by PS. As observed in a previous study, the growth inhibition of marine algae could recover from exposure to PS [46], which was consistent with this study. After recovery for 96 h, the algal density of PS and PMMA groups was increased by 1.13–10.59% and 5.67–7.76%, respectively (Figure 1c). The enhancement of algal growth after recovery represented a stress-response hormesis, an effect that is often described as beneficial to organisms with appropriate stress while harmful at a higher intensity [49,50].

The photosynthetic pigments Chl a and c are responsible for capturing solar energy, and play vital roles in the photosynthesis of diatoms [51,52]. In Figure 1d, PS and PMMA decreased the Chl a content by 8.01–12.17% and 0.59–12.56%, respectively, which was consistent with the inhibition of algal density in Figure 1c. After recovery, Chl a content in PS and PMMA groups was still lower than that in the control group by 8.38–22.91% and 11.73–19.83%, respectively (Figure 1d). This suggested that MP exposure had persistent negative effects on the biosynthesis of Chl a in algae. After exposure, no significant difference was observed between MPs and control groups in the Chl c content, as well as in the recovery stage (Figure 1e). After recovery, the growth of algae was recovered in Figure 1c, while the biosynthesis of Chl a held persistent impairment. This suggested that algae may compensate growth through other ways, such as the in vitro uptake of nutrient elements [22,53,54]. The non-recoverability of Chl a synthesis was related to the damage of chloroplasts, as observed in the TEM images of algae below.

Carotenoids, which have photoprotective functions, are generally found in diatoms [51]. As shown in Figure 1f, PS and PMMA reduced the carotenoid content in algae by 0.65–12.42% and 9.15–19.61% more than the control, respectively. After recovery, there was no significant difference between the carotenoid content in control and MP treatment groups (Figure 1f), suggesting that the carotenoid biosynthesis in algae exposed to MPs was recoverable. Chloroplast damage caused by MPs potentially affected Chl a biosynthesis for a long time, reducing photosynthesis and thus weakening the primary productivity of diatoms [35].

### 3.2. Cellular Oxidative Stress Response to MPs

**Figure 2:**
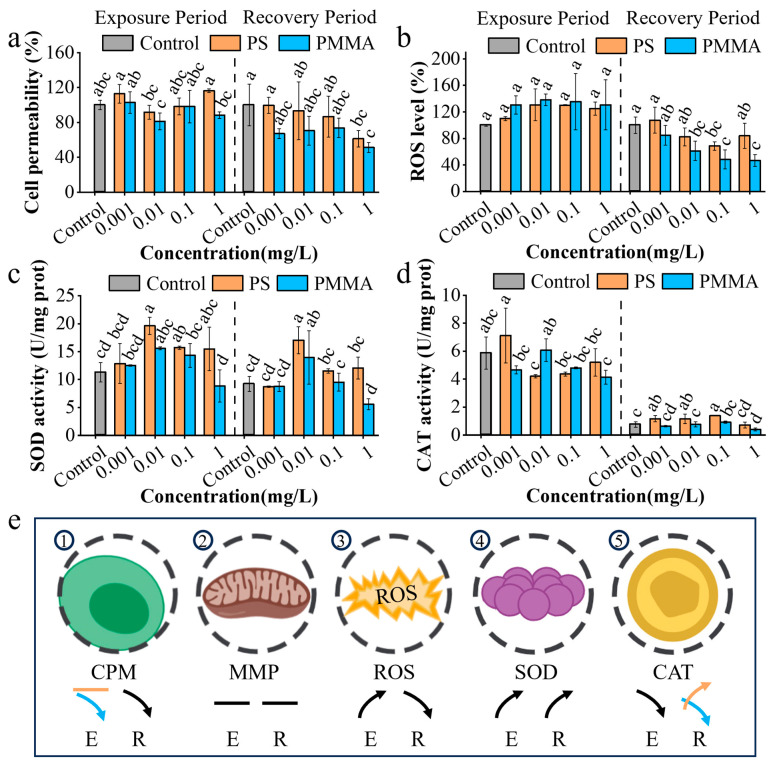
Cellular oxidative stress responses to MPs. (**a**) Relative cell permeability. (**b**) Relative ROS level. (**c**) SOD activity. (**d**) CAT activity. (**e**) Illustration of oxidative stress responses of algae after exposure and recovery. E, exposure period; R, recovery period. The orange arrows represent PS, the blue arrows represent PMMA, and the black arrows represent both PS and PMMA. The upward- and downward-facing arrows indicate upregulation and downregulation, respectively, and the horizontal bars indicate no significant change. The different lowercase letters in the figure denote significant differences, *p* < 0.05.

Changes in cell permeability induced by oxidative stress can reflect damage to the algal cell wall and plasma membrane [50,55]. After exposure, PS had no significant effects on the cell permeability of algae, while PMMA reduced cell permeability by 1.69–18.79% at 0.01–1 mg/L compared with the control (Figure 2a). The reduction in cell permeability with PMMA exposure was similarly found in *Phaeodactylum tricornutum* in zeolitic imidazolate framework (ZIF) exposure [37], indicating a defense mechanism that blocks MPs from entering cells. After recovery, the cell permeability in the PS and PMMA groups was decreased by 0.56–38.6% and 26.13–48.67%, respectively (Figure 2a). The decrease in cell permeability was also observed in *Chlorella vulgaris* recovered from graphene oxide quantum dots (GOQDs) [50]. The decreased cell permeability was associated with the regulation of related gene expression and metabolic activity [56], reflecting a defense mechanism in cells. Compared with the control, MPs had no significant influence on mitochondrial membrane potential in algae in the exposure and recovery stages (Figure S4).

As shown in Figure 2b, ROS levels in PS and PMMA groups were increased by 10.06–30.51% and 30.46–38.12%, respectively (Figure 2b), suggesting the oxidative stress in algae induced by MPs. Similar results of an increase in ROS were observed in a diatom exposed to PS acclimation [35]. After recovery, ROS levels in PS and PMMA groups were reduced by 16.09–31.41% and 15.34–53.13%, respectively, compared with the control (Figure 2b). Studies have shown that the quenching of free radicals such as OH in algal cells explained the decrease in ROS levels [37,57], which contributed to the complete ROS scavenging systems in algae [58]. The decrease in ROS levels after recovery suggested an attenuation of oxidative stress that weakened the damage to the ultrastructure of algae and promoted algal growth during the recovery period (Figure 1c).

SOD catalyzes the disproportionation of superoxide anions, generates H_2_O_2_ and O_2_, and prevents the oxidative damage of excessive superoxide anions, which plays an important role in the biological antioxidant system [59]. The results in Figure 2c show that the SOD activity induced by PS and PMMA was increased by 14.01–73.94% at 0.001–1 mg/L and 10.9–38.03% at 0.001–0.1 mg/L, respectively, suggesting that the algal defense mechanism was activated. After recovery, the SOD activity bore a similarity to that in the exposure period (Figure 2c), suggesting the persistent oxidative stress responses to MP exposure.

CAT is a critical enzyme for removing H_2_O_2_ in cells, and plays an important role in the ROS scavenging system [60]. Compared to the control, PS and PMMA exposure inhibited CAT activity by 11.26–28.33% and 18.09–29.52%, respectively (Figure 2d), which supported the increase in ROS levels in the exposure period in Figure 2b. After recovery, the CAT activity induced by PS was increased by 55.41–86.49%, indicating the recovery from CAT activity inhibition, which reflected the stress-response hormesis [49,50]. Diatom cells are at different growth stages during the exposure and recovery stages, which could have induced changes in the control group at exposure and recovery periods. Due to the algal self-defense mechanism at the PS recovery stage, excessive ROS in cells was effectively eliminated, thus alleviating the toxicity of MPs [61]. The persistent inhibition of activity was observed in PMMA groups (14.86–47.3%) (Figure 2d), indicating the persistent toxicity of PMMA on CAT. The above algal oxidative stress responses to MPs are illustrated in Figure 2e.

### 3.3. Damage of Cellular Ultrastructure

**Figure 3:**
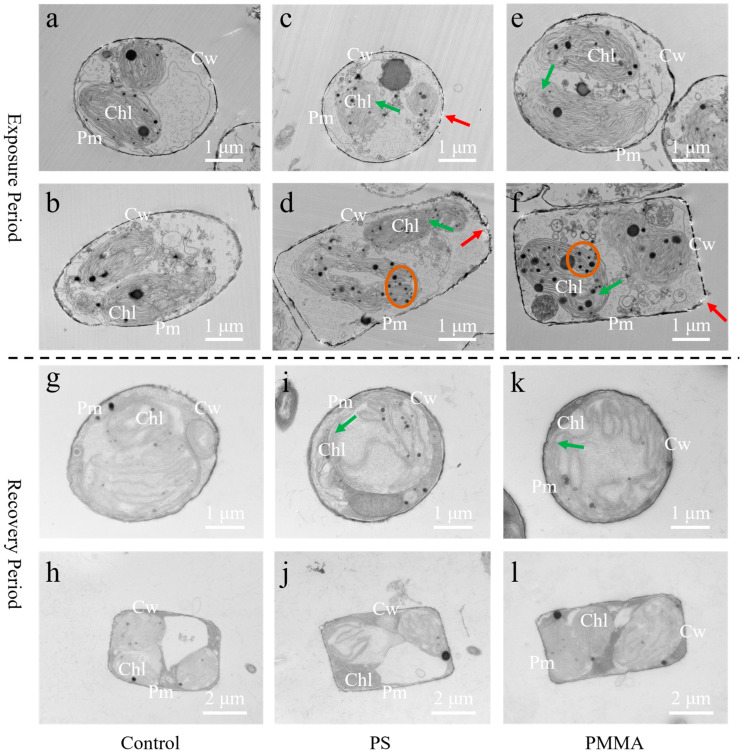
TEM images of diatoms at 96 h of exposure and recovery. (**a**–**f**) TEM images of different sections of diatoms in the exposure period. (**g**–**l**) TEM images of different sections of diatoms in the recovery period. The red and green arrows denote damage to cell walls and chloroplasts, respectively. The orange circles denote starch grains. Cw, cell wall; Pm, plasma membrane; Chl, chloroplast.

The SEM image showed that the microalgal cell size was approximately 3–5 μm and had a porous surface structure (Figure S5). TEM images showed that the cell walls, plasma membranes, chloroplasts, and other cytoplasmic compartments were clearly visible in control groups (Figure 3a,b). PS and PMMA exposure could damage the ultrastructure of microalgae, such as the damage to cell walls (red arrows) and chloroplast integrity (green arrows) in Figure 3c–f. Compared to the control, PS and PMMA increased cell wall damage by 25.56% and 20%, respectively (Figure S6). Similar cell wall damage was also observed in freshwater algae exposed to PS nanoplastics (NPs) in a previous study [62]. The chloroplast blurring caused by PS and PMMA was increased by 14.24% and 12.28%, respectively (Figure S6), which was consistent with the decrease in Chl a in Figure 1. In addition, a higher number of starch grains was observed in microalgal cells after PS and PMMA exposure compared to the control (orange circles) in Figure 3d–f. A similar phenomenon has been found in freshwater and marine microalgae exposed to PS MPs and graphene oxide in previous studies [63,64]. The increased number of starch grains in microalgal cells is considered a defense mechanism to resist and reduce the damage of MPs [65]. After recovery, the microalgal cell walls in PS and PMMA groups were clear and intact, similar to that of the control (Figure 3g–l). However, the chloroplast blurring in PS and PMMA groups (green arrows in Figure 3i,k) was still increased by 13.19% and 11.89%, respectively (Figure S6), indicating that the chloroplast damage was difficult to recover, consistent with the decrease in Chl a after recovery in Figure 1. Sustained chloroplast damage potentially affected photosynthetic activity, thereby reducing the primary productivity of diatoms [35].

### 3.4. Effects of MPs on Nitrogen Assimilation

**Figure 4:**
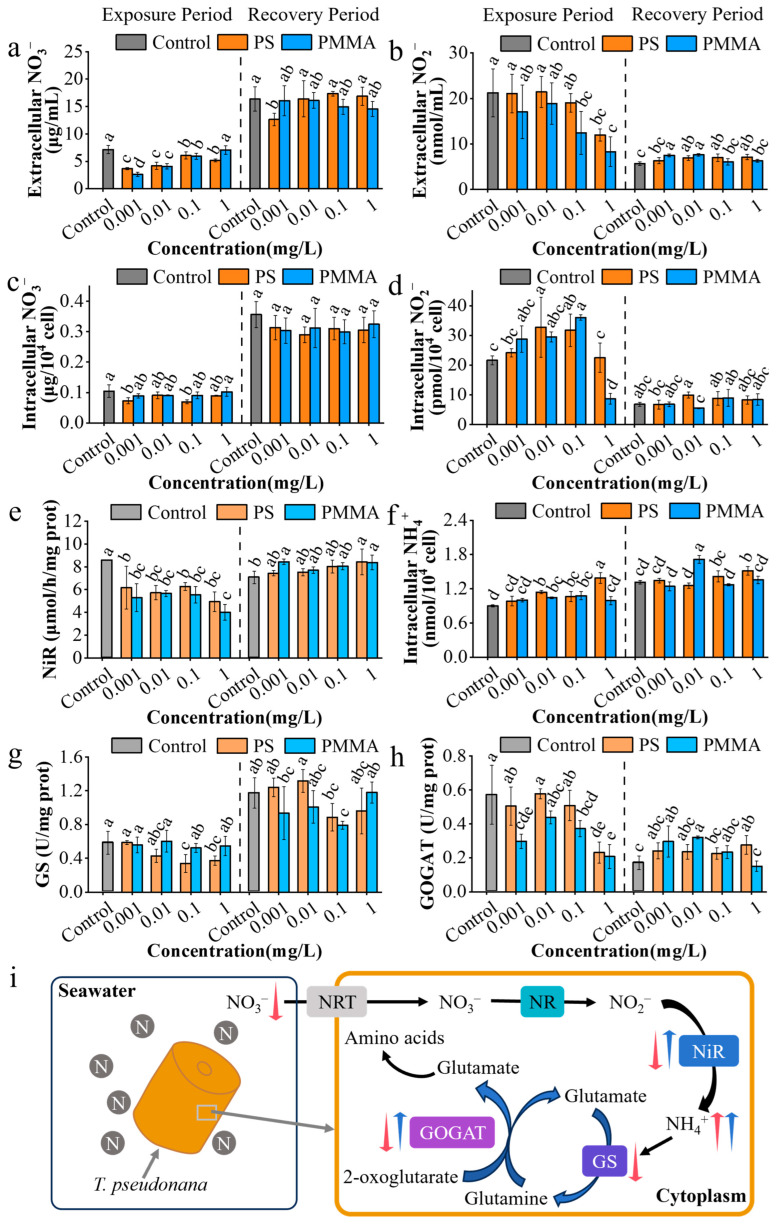
Effects of MPs on nitrate utilization in *T. pseudonana*. (**a**,**b**) Extracellular nitrate and nitrite contents. (**c**,**d**) Intracellular nitrate and nitrite contents. (**e**) NiR activity. (**f**) Intracellular ammonium content. (**g**,**h**) GS and GOGAT activities. (**i**) Schematic plot of nitrate utilization affected by MPs after exposure and recovery. NRT, nitrate transporters. The red and blue arrows indicate the exposure and recovery periods, respectively. The upward- and downward-facing arrows indicate upregulation and downregulation, respectively. The different lowercase letters in the figure denotes significant differences, *p* < 0.05.

As shown in Figure 4a, the extracellular nitrate concentrations in the media after PS and PMMA exposure were significantly reduced by 15.18–48.85% and 1.31–63.36%, respectively. The decrease in nitrate concentration in the media was related to the intake of algae facilitated by MPs [35]. The extracellular nitrate concentration was observed to return to a similar level to the control in the recovery stage (Figure 4a). The extracellular nitrite concentrations of PS and PMMA were decreased by 0.75–43.6% and 3.86–69.44% compared to the control, respectively, with a significantly decrease at 1 mg/L (Figure 4b), suggesting a similar nitrogen intake to nitrate [35]. The extracellular nitrite concentrations after PS and PMMA recovery were increased by 11.27–25.18% and 7.04–34.33%, respectively (Figure 4b), which were related to the excretion of nitrite in cells.

The protein content induced by PS and PMMA had no significant change in the exposure period but increased after recovery in the PMMA groups (Figure S7a). The phenomenon of an increase in protein content was considered a stress-response hormesis by enhancing the utilization of nitrogen by algae, promoting protein synthesis, and thus increasing the protein contents in algae [35,49,50]. As shown in Figure S7b, no significant difference was observed between the control and MP treatment groups on NR activity in the exposure stage. After recovery, the NR activity of PS and PMMA was increased by 29.04–60.62% and 1.24–28.37%, respectively (Figure S7b). The increase in NR activity bore a similarity to that of protein content, which showed stress-response hormesis [49,50]. After exposure, the intracellular nitrate contents of PS and PMMA were decreased by 13.33–34.29% and 2.86–15.24%, respectively (Figure 4c), and the intracellular nitrite contents of PS and PMMA were increased by 3.96–51.59% and 32.96–66.57%, respectively (Figure 4d), indicating the accumulation of nitrite in cells. After recovery, nitrate levels in MP-treated groups returned to the levels of controls (Figure 4c,d), indicating the recoverability of nitrate levels in algal cells.

Nitrite reduction to ammonium is catalyzed by NiR in the chloroplast matrix, and ammonium nitrogen is a key precursor for conversion to nitrogen-containing amino acids [66,67]. Exposure to PS and PMMA significantly reduced the NiR activity by 26.89–42.44% and 34.02–53.24%, respectively (Figure 4e). The decreased NiR activity caused low reduction efficiency and accumulation of nitrite in cells, supporting the increase in nitrite content after exposure in Figure 4d. However, the recovery of NiR activity was observed in the recovery period (Figure 4e). After exposure, the ammonium nitrogen levels of PS and PMMA in algal cells were increased by 9.23–54.73% and 10.23–19.91%, respectively (Figure 4f). Similarly, after recovery, the ammonium nitrogen levels were increased by 2.29–15.27% and 3.82–31.3% in PS and PMMA groups, respectively (Figure 4f). The above promotion phenomenon can be conducive to the synthesis of amino acids to get more involved in cell metabolism, and thus promoted the growth of algae [53].

GS and GOGAT are key enzymes in ammonium assimilation in cells, which together constitute the GS/GOGAT cycle that eventually embeds ammonium in the carbon skeleton in the form of glutamate [66]. Exposure to PS inhibited the GS activity by 26.63–42.1%, while no significant change was presented after PMMA exposure compared with the control (Figure 4g), indicating the inhibitory effects of PS on GS activity in algae. After recovery, GS activity was observed to return to a level similar to that in the control (Figure 4g). Similarly, after exposure, the GOGAT activity of algae in PS and PMMA groups was decreased by 11.21–59.54% and 23.47–63.75%, respectively (Figure 4h). The similar reduction in GOGAT activity was also presented in *Chaetoceros gracilis* exposed to PS MPs [54]. The recovery of GOGAT activity was observed in the recovery period (Figure 4h) and was consistent with that of NR and NiR activity. The enhancement of the GS/GOGAT cycle after recovery improved the availability of ammonium, and thus promoted the protein synthesis (Figure S7a), resulting in higher cell density in the recovery period (Figure 1c). The presence of MPs changed the nitrogen storage forms and assimilation processes in *T. pseudonana*, as illustrated in Figure 4i. MP exposure reduced NiR, GS, and GOGAT activities and caused low efficiency of the GS/GOGAT cycle, which affected amino acid biosynthesis. However, the activities of enzymes related to nitrogen assimilation were recovered in the recovery period, and the nitrogen cycle of diatoms returned to control levels, and had even strengthened.

### 3.5. Mechanisms of Algal Toxicity and Recovery

**Figure 5:**
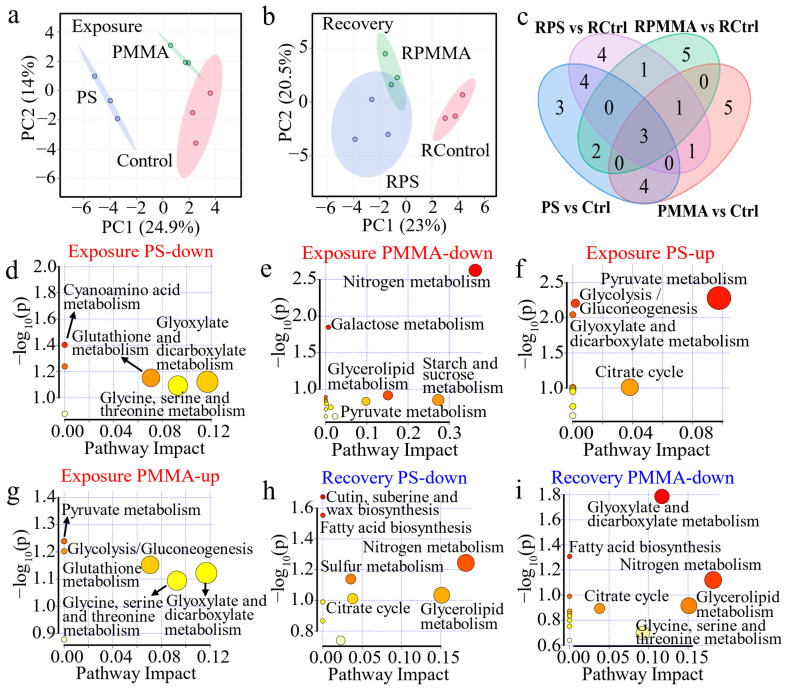
Metabolic analysis of algae at 96 h in the exposure and recovery periods. (**a**,**b**) PLS-DA of metabolites. (**c**) Veen map of DEMs. (**d**–**i**) Metabolic pathway perturbation. The color and size of the circles indicate the significance and degree of influence of the metabolic pathway, respectively. The darker the color and the larger the size of the circle, the greater the significance and influence of the metabolic pathway.

As shown in Figure S8, approximately 50 metabolites in algal cells were identified by GC-MS, including fatty acids, amino acids, carbohydrates, and other small-molecule substances. Partial least squares-discriminant analysis (PLS-DA) indicated that both PS and PMMA interference in metabolism was presented in the exposure stage (Figure 5a), and the persistent interference of both MPs in metabolism was observed in the recovery stage (Figure 5b). In the exposure stage, there were 16 DEMs with PS exposure and 14 DEMs with PMMA exposure, and 7 DEMs were co-regulated by both MPs (Figure 5c). After recovery, PS and PMMA regulated 14 and 12 DEMs, respectively, of which 5 DEMs were co-regulated by both (Figure 5c).

The pathway perturbation of these DEMs was analyzed by Kyoto Encyclopedia of Genes and Genomes (KEGG) pathway analysis. After PS exposure, glyoxylate and dicarboxylate metabolism, glycine, serine and threonine metabolism, and glutathione metabolism were downregulated (Figure 5d). The glyoxylate cycle plays an important role in gluconeogenesis, establishing a connection between the P-oxidation of fatty acids and enzymes that convert fat to carbohydrates, which has great significance for supporting algal growth and maintaining marine primary productivity [68,69]. The downregulation of glyoxylate and dicarboxylate metabolism supported the decrease in the algal density of the PS group in Figure 1. It is reported that glycine, serine, and threonine metabolism regulated the cell membrane, and was closely related to glutathione metabolism, which served as a portion of the antioxidant system in cells, and the reduced glycine was responsible for the downregulation of glutathione metabolism [70,71]. Dissolved inorganic nitrogen (e.g., NO_3_^−^ and NH_4_^+^) is assimilated by organisms as the main component of nitrogen-containing organic matter, and participates in nitrogen metabolism [72]. Alterations in the nitrogen metabolism of diatoms have profound and lasting implications for the community structure and marine biogeochemical cycles [73]. PMMA downregulated nitrogen metabolism (Figure 5e), confirming the reduced enzymatic activities related to nitrogen assimilation. Galactose metabolism and starch and sucrose metabolism were also downregulated (Figure 5e), suggesting the energy inhibition of PMMA on algae. PS and PMMA co-upregulated pyruvate metabolism and glycolysis/gluconeogenesis (Figure 5f,g), indicating that algal cells responded to stress by increasing energy production. In addition, PS upregulated citrate cycle (TCA cycle), explaining that algae responded to PS exposure by enhancing carbohydrate catabolism, as reported in a former study [35]. PMMA upregulated glycine, serine, and threonine metabolism, and glutathione metabolism (Figure 5g), which exhibited a close connection to the decrease in cell permeability in Figure 2a [37].

The down-regulated pathway at the recovery stage of PS was shown in Figure 5h, showing that the effect of glutathione metabolism disappeared. The down-regulated galactose metabolism of the PMMA group was restored after recovery in Figure 5i; however, the glycine, serine, and threonine metabolism was downregulated, which was associated with the decrease in cell permeability in the PMMA recovery period (Figure 2a). Although the activities of enzymes involved in nitrogen assimilation were recovered from PS and PMMA exposure (Figure 4), the nitrogen metabolism experienced significant downregulation (Figure 5h,i). The carbon fixed by photosynthesis in algae is first used in chloroplasts to synthesize fatty acids [58]. Fatty acid biosynthesis was downregulated in the PS and PMMA recovery stages, coincident with the decrease in Chl a (Figure 1) experienced in photosynthesis. As shown in Figure S9a,b, glyoxylate and dicarboxylate metabolism, pyruvate metabolism, and glycolysis/gluconeogenesis at the recovery stage of PS and PMMA were upregulated, resulting in enhanced algal growth in the recovery period in Figure 1c [74,75]. Pantothenate and coenzyme A (CoA) play vital roles in the synthesis and oxidation of fatty acids. The upregulation of pantothenate and CoA biosynthesis in the PMMA recovery stage partly compensated for the downregulation of fatty acid biosynthesis, increased the degree of unsaturation of algal fatty acids, and then reduced ROS production (Figure 2b) [76].

**Figure 6:**
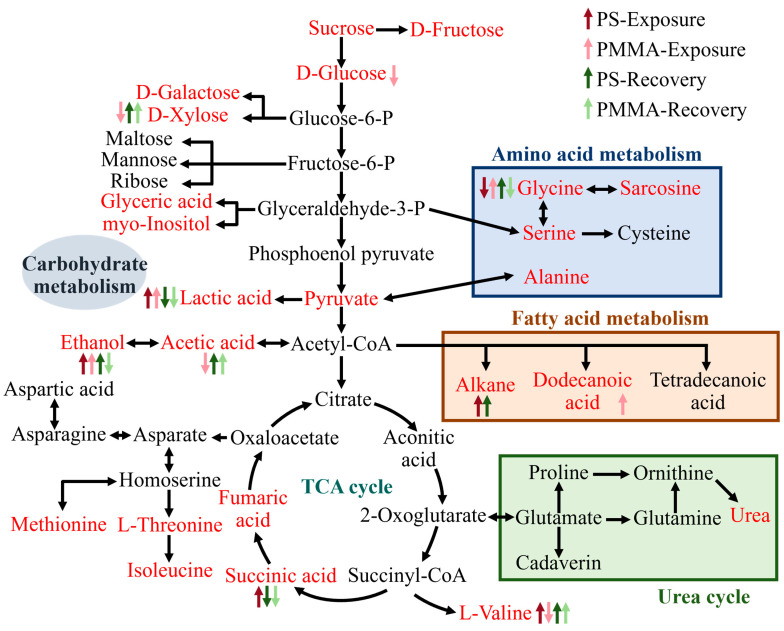
Overview of the phytotoxicity mechanisms of MPs studied by metabolic analysis. The red text denotes metabolites detected in this work. The upward- and downward-facing arrows indicate upregulated and downregulated metabolites, respectively (this figure is created based on the KEGG database and our previous study) [79].

The mechanisms of the toxicity of MPs on algae are displayed in Figure 6. MPs disrupted carbohydrate, amino acid, and fatty acid metabolisms. The glycolysis provides pyruvate, which is decarboxylated and oxidized sequentially to produce acetyl-CoA that participates in the TCA cycle, and promotes energy production and biosynthesis [77]. Algal cells responded to PS exposure by facilitating energy production through the upregulation of glycolysis, fatty acid metabolism, and TCA cycle (e.g., lactic acid, alkane, and succinic acid). However, the energy metabolism of algae was hindered after PMMA exposure (e.g., the downregulation of d-glucose, d-xylose, and acetic acid), yet the upregulation of lactic acid and dodecanoic acid compensated for energy deficits as defensive strategies [78]. After recovery, the metabolic disturbance involved in carbohydrate metabolism in the PS and PMMA groups persistently existed, indicating that routine metabolic perturbations in algae were uneasy to recover. The above metabolomics analysis results provided insights into the mechanisms of the persistence and recovery of MP toxicity.

## 4. Conclusions

This study revealed that PS and PMMA MPs significantly inhibited the growth of *T. pseudonana* at 0.1 mg/L, and the inhibition with PS was easier to recover than PMMA due to the enhancement of carbohydrate metabolisms (e.g., glyoxylate and dicarboxylate metabolism, pyruvate metabolism, and glycolysis/gluconeogenesis). PS and PMMA induced oxidative stress with the increase in ROS levels in microalgae. In addition, PS and PMMA induced cell ultrastructural damage, and the persistent inhibition of Chl a in PS and PMMA groups was attributed to the persistent chloroplast blurring. Moreover, PS and PMMA significantly inhibited the activity of nitrogen assimilation enzymes at 1 mg/L, and although the inhibition was recoverable at the recovery stage, the nitrogen metabolism was still down-regulated. In general, compared with PMMA, PS induced significant inhibition of algal growth, while no significant differences were found in other toxicity indicators. These findings highlight the complexity of the persistence and recovery of MP phytotoxicity and contribute to a better understanding and assessment of the environmental risks posed by MPs, particularly their effects on nitrogen assimilation in diatoms. Moreover, this study supports future applications of biotechnology, including the following: (1) establishing a standardized toxicity testing system using diatoms to evaluate the physiological and genotoxic effects of MPs on organisms; (2) developing environmentally friendly pollutant treatment programs by screening microalgae capable of degrading MPs; and (3) alleviating the toxic effects of MPs on marine organisms by regulating the metabolic pathways of diatoms.

## Data Availability

The data that support the findings of this study are available from the corresponding author upon reasonable request.

## Supplementary Materials

The following supporting information can be downloaded at: https://www.mdpi.com/article/10.3390/toxics13050376/s1, Figure S1: Relationship between algal density and absorbance; Figure S2: Characterization of MPs; Figure S3: Persistence and recovery of algal growth to MPs; Figure S4: Mitochondrial membrane potential (MMP) in algae after 96 h of MPs exposure and recovery; Figure S5: Representative SEM of *T. pseudonana*; Figure S6: Statistical analysis of cell wall and chloroplast damage in diatoms in the TEM images at 96 h; Figure S7: Protein content and Nitrate reductase (NR) activity; Figure S8: Heat map of the metabolites; Figure S9: Up-regulated metabolic pathways in PS and PMMA groups in the recovery period; Table S1: Components of the F/2 medium.
