# Supplementary material for: Persistence and Recovery of Polystyrene and Polymethyl Methacrylate Microplastic Toxicity on Diatoms
Source: Toxics. 2025 May 6;13(5):376. doi: 10.3390/toxics13050376 (PMC12115889; doi:10.3390/toxics13050376)
Supplement: Supplementary file 1 [file toxics-13-00376-s001.zip › toxics-3575452-supplementary.pdf]

Supplementary materials

Table S1. Components of the F/2 medium.

Component	Solution concentration
$NaNO_3$	75 g/L
$NaH_2PO_4 \cdot H_2O$	5 g/L
$Na_2SiO_3 \cdot 9H_2O$	30 g/L
$FeCl_3 \cdot 6H_2O$	3.15 g/L
$Na_2EDTA \cdot 2H_2O$	4.36 g/L
$CuSO_4 \cdot 5H_2O$	9.8 mg/L
$Na_2MoO_4 \cdot 2H_2O$	6.3 mg/L
$ZnSO_4 \cdot 7H_2O$	22 mg/L
$CoCl_2 \cdot 6H_2O$	10 mg/L
$MnCl_2 \cdot 4H_2O$	180 mg/L
Vitamin B12	1 mg/L
Biotin	1 mg/L

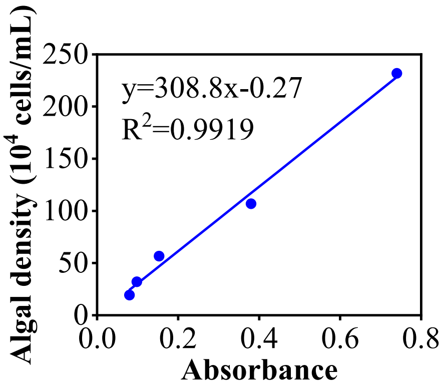

Figure S1. Relationship between algal density and absorbance.

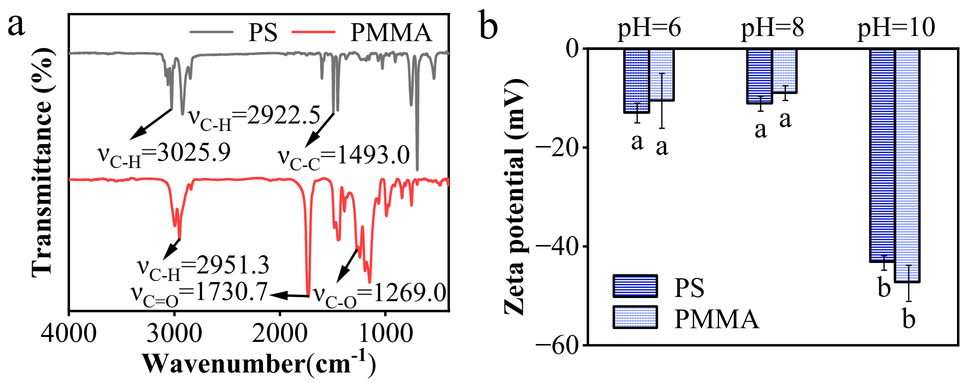

Figure S2. Characterization of MPs. (a) FTIR spectra of MPs. (b) Zeta potential of MPs in ultrapure water with pH values of 6, 8, and 10, respectively.

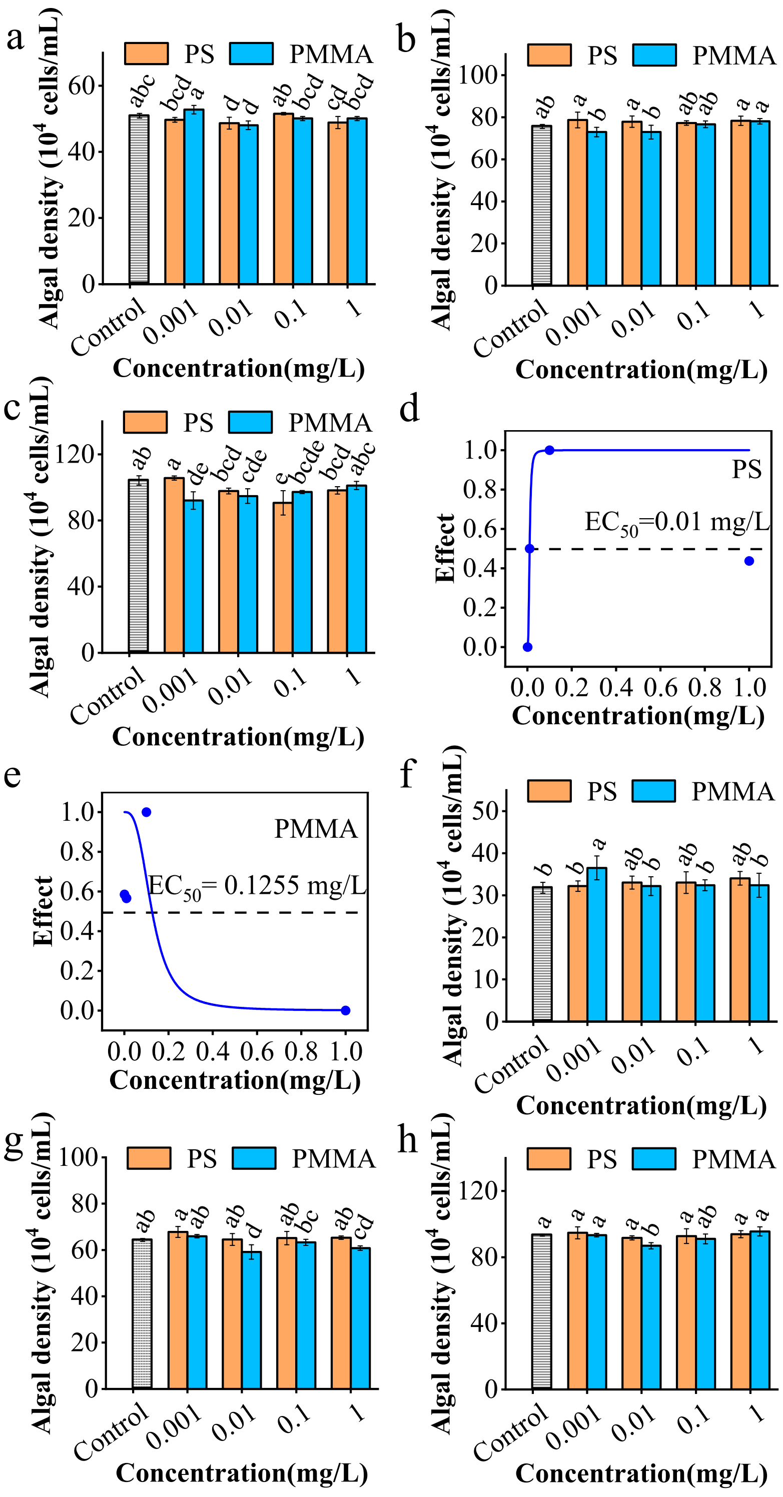

Figure S3. Persistence and recovery of algal growth to MPs. (a-c) Algal densities after 24, 48, 72 h of MPs exposure. (d, e) EC_{50} of PS and PMMA at 96 h. (f-h) Algal densities after 24, 48, 72 h of MPs recovery. The different lowercase letters in the figure denotes significant differences, $p < 0.05$.

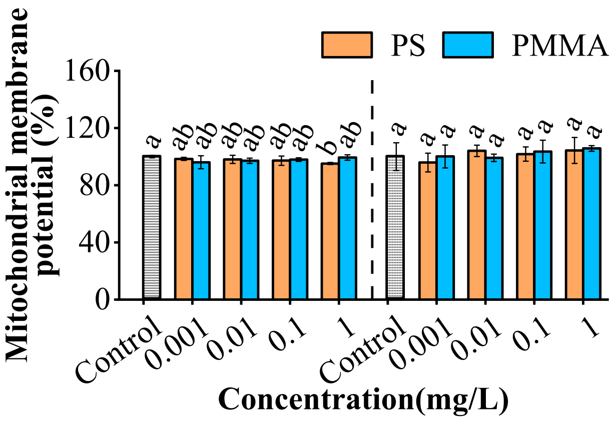

Figure S4. Mitochondrial membrane potential (MMP) in algae after 96 h of MPs exposure and recovery. The different lowercase letters in the figure denotes significant differences, $p < 0.05$.

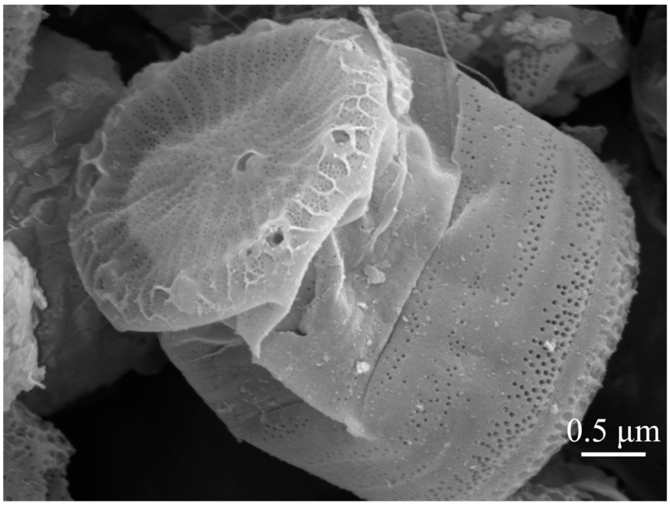

Figure S5. Representative SEM of *T. pseudonana*.

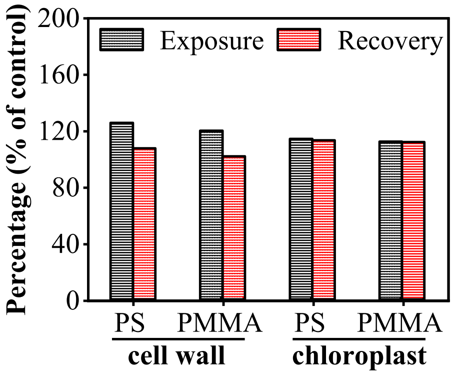

Figure S6. Statistical analysis of cell wall and chloroplast damage in diatoms in the TEM images at 96 h.

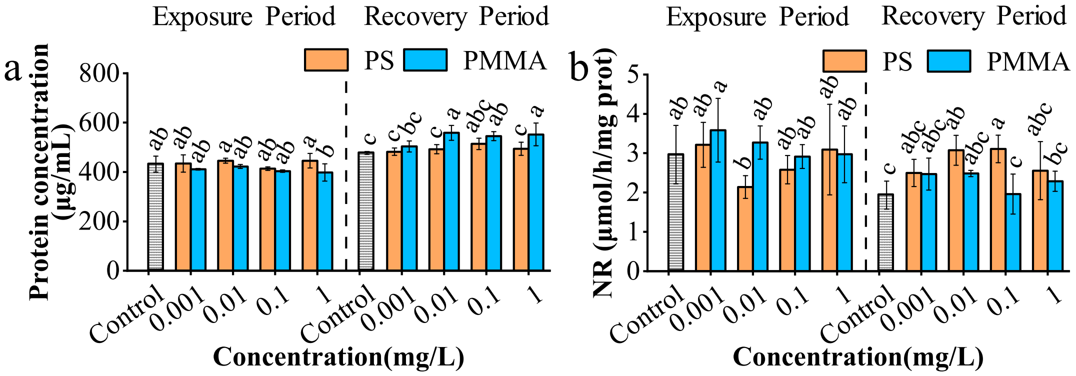

Figure S7. (a) Protein content. (b) Nitrate reductase (NR) activity. The different lowercase letters in the figure denotes significant differences, $p < 0.05$.

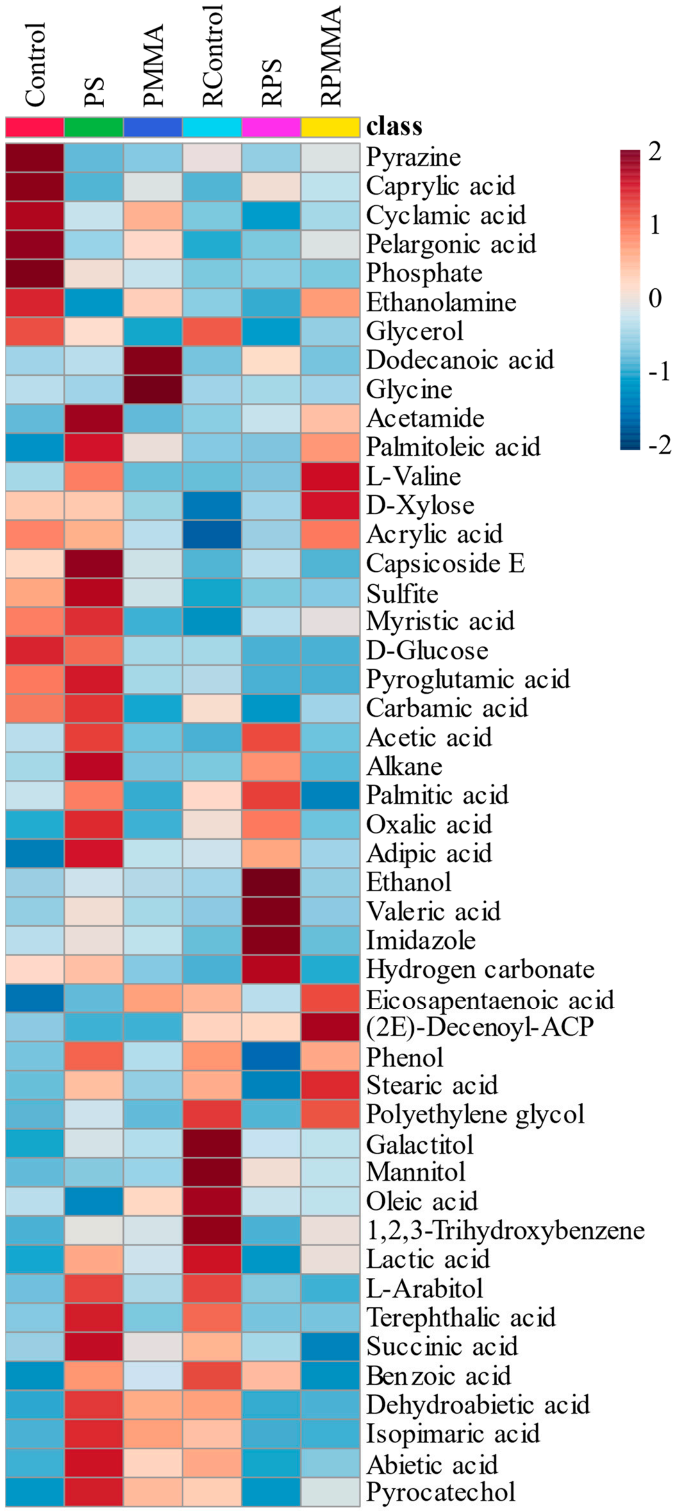

Figure S8. Heat map of the metabolites.

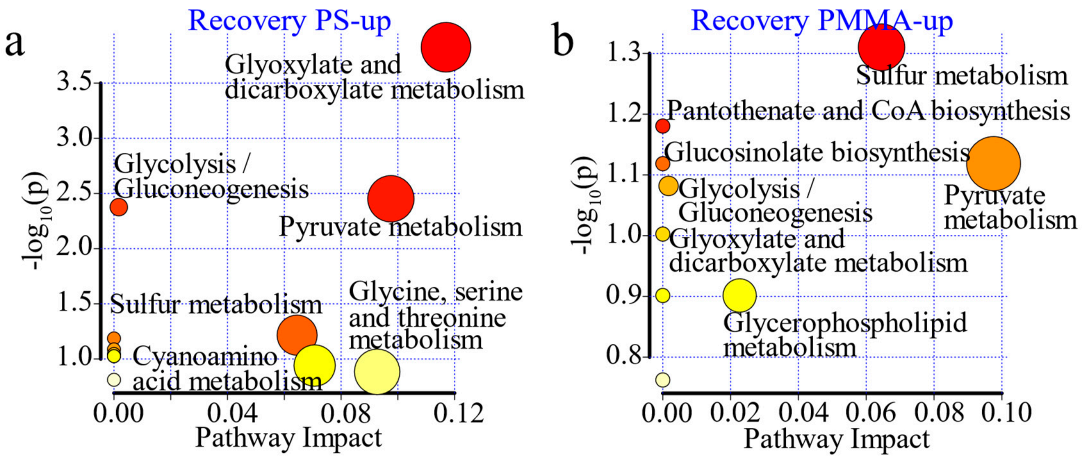

Figure S9. Up-regulated metabolic pathways in PS (a) and PMMA (b) groups in the recovery period.
